# Quantitative Analysis of Intracellular Ca^2+^ Release and Contraction in hiPSC-Derived Vascular Smooth Muscle Cells

**DOI:** 10.1016/j.stemcr.2019.02.003

**Published:** 2019-03-07

**Authors:** Oleh V. Halaidych, Amy Cochrane, Francijna E. van den Hil, Christine L. Mummery, Valeria V. Orlova

**Affiliations:** 1 Department of Anatomy and Embryology, Leiden University Medical Center, Einthovenweg 20, 2333 ZC Leiden, The Netherlands

**Keywords:** human induced pluripotent stem cells (hiPSCs), vascular smooth muscle cells (vSMCs), neural crest-derived vascular smooth muscle cells (NC-SMCs), real-time intracellular Ca^2+^ release in vSMCs, microfluidics, contraction, cell tracking, automated image analysis, CellProfiler, LC_Pro plugin for ImageJ

## Abstract

Vascular smooth muscle cells (vSMCs) are highly heterogeneous across different vascular beds. This is partly dictated by their developmental origin but also their position in the vascular tree, reflected in their differential responses to vasoactive agonists depending on which arteriolar or venular segment they are located. Functional assays are necessary to capture this heterogeneity *in vitro* since there are no markers that distinguish subtypes. Here we describe methods for determining real-time intracellular Ca^2+^ release and contraction in vSMCs of neural crest origin differentiated from human induced pluripotent stem cells using multiple protocols, and compare these with primary human brain vascular pericytes and smooth muscle cells. Open-source software was adapted for automated high-density analysis of Ca^2+^-release kinetics and contraction by tracking individual cells. Simultaneous measurements on hundreds of cells revealed heterogeneity in responses to vasoconstrictors that would likely be overlooked using manual low-throughput assays or marker expression.

## Highlights

• Microfluidic assay for real-time Ca^2+^ release in hiPSC-derived vSMCs
• Automated Ca^2+^ release and contraction analysis in hiPSC-derived vSMCs
• Side-by-side comparison of hiPSC-derived vSMCs with human brain pericytes and vSMCs

## Graphical Abstract

**Figure:**
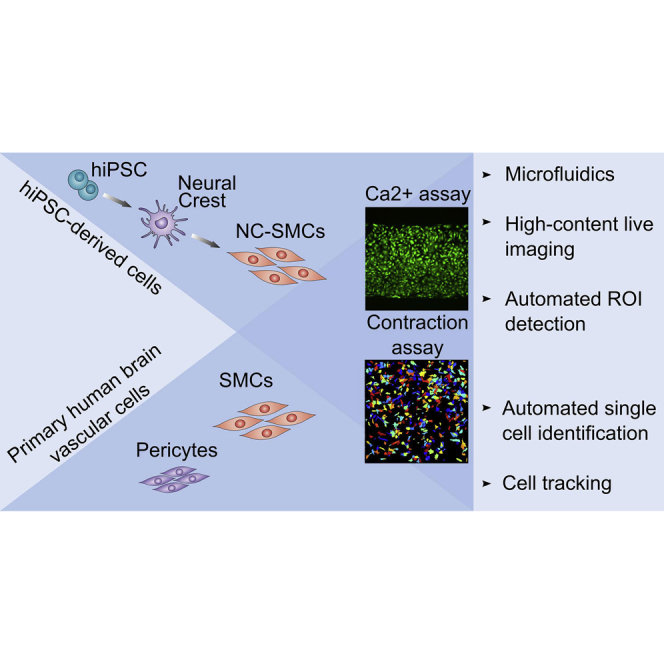

## Teaser

In this article, Orlova and colleagues describe methods for real-time intracellular Ca^2+^ release and contraction in vascular smooth muscle cells differentiated from human induced pluripotent stem cells. Open-source software adapted for automated high-density analysis and simultaneous measurements of hundreds of cells revealed heterogeneity in responses to vasoconstrictors that would likely be overlooked using standard low-throughput assays or marker expression.

## Introduction

Vascular smooth muscle cell (vSMC) dysfunction is associated with many diseases ranging from atherosclerosis and hypertension to cerebrovascular disorders (Owens et al., 2004, Sinha and Santoro, 2018). Developmentally, vSMCs originate from multiple lineages including mesoderm and neural crest, the primary source of vSMCs in the cerebral vasculature (Majesky, 2007). Human induced pluripotent stem cells (hiPSCs) have been shown to be an excellent source of vSMCs of various developmental origins (Cheung et al., 2014, Cheung et al., 2012, Granata et al., 2017), presenting new opportunities for disease modeling and drug discovery using patient-specific cells. Despite improvements in protocols for vSMC differentiation, assays for their automated functional characterization have lagged behind. vSMCs, like striated cardiac and skeletal myocytes, are contractile and their contractile responses are correlated with global changes in intracellular Ca^2+^ (Wray et al., 2005). vSMCs *in situ* exhibit rapid intracellular Ca^2+^ release in response to vasoconstrictors. The initial response occurs within ∼10 s and is followed by prolonged wave-like oscillations as a result of intracellular Ca^2+^ release and reuptake (Borysova et al., 2013). Cultured vSMCs exhibit profound heterogeneity in their responses to vasoconstrictors. However, most studies of intracellular Ca^2+^ release do not capture this heterogeneity in responses. Intracellular Ca^2+^ release is typically measured as a low-throughput assessment of selected regions of interest that may not represent the whole cell population and its intrinsic heterogeneity. In addition, even though the flow-cytometry method to determine intracellular Ca^2+^ is high-throughput, it lacks physiological relevance and does not allow tracking of Ca^2+^ flux in individual cells over time so that signal shape parameters cannot be derived. High-throughput, robust, and standardized assays that accurately assess vSMC functionality would be of value in monitoring drug responses and disease phenotypes.

Here, we developed functional assays and an automated quantification framework for intracellular Ca^2+^ release and contraction in vSMCs. vSMCs were differentiated via neural crest intermediates from three independent healthy hiPSC lines, using various protocols based on previously published methods (Cheung et al., 2012, Dash et al., 2016, Granata et al., 2017, Wanjare et al., 2013, Wanjare et al., 2014). The functionality of these hiPSC-derived vSMCs was compared side by side with primary human brain vascular pericytes (HBVPs) and human brain vSMCs (HBVSMCs) using a set of well-established vasoconstrictors. Heterogeneity in responses of both hiPSC-derived and primary vSMCs was observed that would likely be overlooked using manual low-throughput assays.

## Results

### Differentiation of Neural Crest Cells from hiPSCs

**Figure 1:**
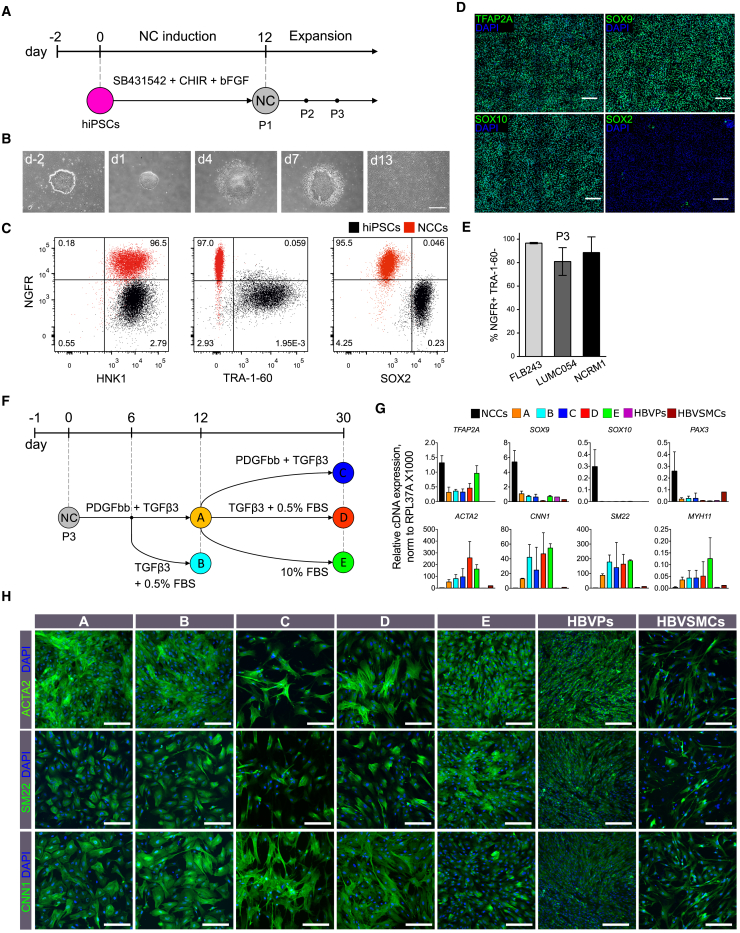
Differentiation of NCCs and NC-SMCs from hiPSCs (A) Schematic illustration of the NCC differentiation protocol. (B) Phase-contrast images of hiPSCs and NCCs at different stages of differentiation. Scale bar, 200 μm. (C) Representative FACS plots showing NGFR, HNK1, TRA-1-60, and SOX2 expression in NCCs at passage 3 (red) and non-differentiated hiPSCs (black). (D) Representative immunofluorescent images showing expression of TFAP2A, SOX9, SOX10, and SOX2 (green), and DAPI (blue) in NCCs. Scale bars, 200 μm. (E) Percentages of NGFR^+^/TRA-1-60^−^ NCCs at passage 3 (P3), differentiated from three hiPSC lines (FLB243, LUMC054, and NCRM1). Error bars are shown as mean ± SD from three independent differentiation experiments. (F) Schematic representation of NC-SMC differentiation. Five conditions are depicted: protocol A (TGF-β3, 2 ng/mL and PDGF-BB, 10 ng/mL for 12 days) (A, yellow), protocol B (TGF-β3, 2 ng/mL and PDGF-BB, 10 ng/mL for 6 days followed by TGF-β3, 1 ng/mL and 0.5% fetal bovine serum [FBS]) (B, light blue), protocol C (TGF-β3, 2 ng/mL and PDGF-BB, 10 ng/mL for 30 days) (C, dark blue), protocol D (TGF-β3, 2 ng/mL and PDGF-BB, 10 ng/mL for 12 days followed by TGF-β3, 1 ng/mL and 0.5% FBS for 18 days) (D, red), and protocol E (TGF-β3, 2 ng/mL and PDGF-BB, 10 ng/mL for 12 days followed by 10% FBS for 18 days) (E, green). (G) RT-PCR analysis of relative gene expression of NC (*TFAP2A*, *SOX9*, *SOX10*, *PAX3*) and SMC (*ACTA2*, *CNN1*, *SM22*, *MYH11*) markers in NCCs, NC-SMCs differentiated using protocols A to E, HBVPs, and HBVSMCs. Error bars represent mean ± SD from three independent experiments and normalized to housekeeping gene *RPL3*7A (×1,000). (H) Representative immunofluorescent images showing expression of ACTA2, SM22, and CNN1 (green) and DAPI (blue) in NC-SMCs (differentiated using protocols A to E), HBVPs, and HBVSMCs. Scale bars, 200 μm.

Bone morphogenetic protein, WNT, and fibroblast growth factor (FGF) signaling are known to be important for the induction of neural crest cells (NCCs) from human pluripotent stem cells (hPSCs) (Cheung et al., 2014, Fukuta et al., 2014, Hackland et al., 2017, Huang et al., 2016, Lee et al., 2007, Leung et al., 2016, Menendez et al., 2011). Since the efficiency of NCC induction in hiPSCs varies, even in defined medium, we tested several protocols in parallel and found that the combination of the transforming growth factor β (TGF-β) inhibitor SB431542 (10 μM), the small molecular WNT activator CHIR99021 (1 μM), and basic FGF (10 ng/mL) using a protocol adapted from previous work (Cheung et al., 2014, Fukuta et al., 2014) was the most robust in defined BPEL (BSA polyvinylalcohol essential lipids) medium (Ng et al., 2008) (Figures 1A, 1B, and S1A). Fluorescence-activated cell sorting (FACS) analysis on day 12 of differentiation showed that 40%–50% of cells were positive for neural crest markers (NGFR and HNK1) and negative for pluripotency markers (TRA-1-60 and SOX2) (Figures S1B and S1C). Importantly, the proportion of NGFR^+^/HNK1^+^/TRA-1-60^−^/SOX2^−^ cells could be enriched to ∼80%–90% at passage 1 (P1) by simple mechanical elimination of the cells in the center of the colonies. FACS analysis showed that NCCs (NGFR^+^/HNK1^+^/TRA-1-60^−^/SOX2^−^) maintained their phenotype up to P7 (Figure S1D). Nuclear localization of neural crest markers, such as TFAP2A, SOX9, and SOX10, as well as downregulation of SOX2 was confirmed by immunofluorescence (Figure 1D). Successful derivation of NCCs using this method was shown in three different hiPSC lines (FLB243, LUMC054, and NCRM1) with comparable high efficiencies (Figure 1E). Notably, NCCs could be cryopreserved at P3 and used as a cell source for further differentiation into SMCs.

### Differentiation of vSMCs from hiPSC-Derived NCCs

We next differentiated NCCs into vSMCs (NC-SMCs) using previously described protocols, with some modifications as shown in Figure 1F (Cheung et al., 2012, Dash et al., 2016, Granata et al., 2017, Wanjare et al., 2014, Wanjare et al., 2013). We also included HBVPs and HBVSMCs as primary human perivascular cells of the same developmental origin for comparison. Analysis of relative gene expression showed downregulation of neural crest markers, such as *TFAP2A*, *SOX9*, *SOX10*, and *PAX3*, and upregulation of SMC contractile markers such as *ACTA2*, *CNN1*, *SM22*, and *MYH11*, in NC-SMCs compared with NCCs; however, expression of *MYH11* remained relatively low (Figure 1G). Expression of contractile SMC markers was similar in all NC-SMCs independent of the differentiation protocol used and was higher than in primary HBVPs and HBVSMCs (Figure 1G). Expression of contractile proteins was confirmed by immunofluorescence (Figures 1H and S1F).

### Quantitative Assessment of Intracellular Ca^2+^ Release in NC-SMCs and Primary vSMCs

**Figure 2:**
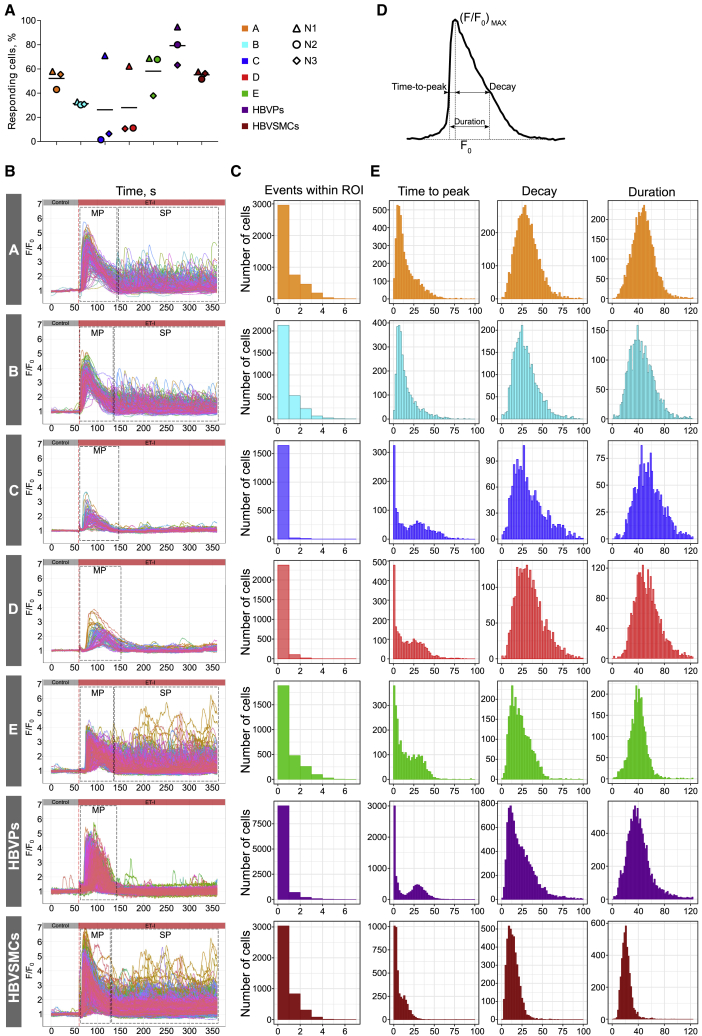
Quantitative Assessment of Intracellular Ca^2+^ Release in NC-SMCs and Primary vSMCs (A) A fraction of responding NC-SMCs (differentiated with protocols A to E), HBVPs, and HBVSMCs stimulated with ET-I (0.1 μM) detected with automated image processing above statistical noise (p < 0.01). Data from three independent experiments are shown. (B) Overlay graph showing normalized average fluorescence intensity F/F_0_ within distinct ROIs over the time of the image sequence measured in NC-SMCs differentiated with protocols A to E, HBVPs, and HBVSMCs. Each individual trace corresponds to one detected and tracked ROI. Red dashed lines indicate a time of stimulation with ET-I (0.1 μM). Black dashed rectangles indicate schematically MPs and SPs. (C) Population histograms of the number of Ca^2+^ release events within each individual ROI in NC-SMCs (differentiated with protocols A to E), HBVPs, and HBVSMCs stimulated with ET-I (0.1 μM). Data from three independent experiments are shown. (D) Schematic representation of the kinetic parameters measured at half-maximum level of normalized fluorescence intensity F/F_0_ used to characterize the shape of the Ca^2+^ peak. (E) Population histograms of MP parameters of Ca^2+^ release (time to peak, decay, and duration) in NC-SMCs (differentiated with protocols A to E), HBVPs, and HBVSMCs stimulated with ET-I (0.1 μM). Data from three independent experiments are shown.

Intracellular Ca^2+^ release was determined in NC-SMCs (differentiated with protocols A to E) and HBVPs and HBVSMCs, using an experimental setup that combined microfluidics and live-cell imaging (Video S1). Fluo-4 was used as an indicator to monitor changes in intracellular Ca^2+^ over time in hundreds of SMCs simultaneously (Figure S2A). Control stimulation was performed using medium only without the addition of the drug (blue box in Figure S2B), followed by drug administration (yellow box in Figure S2B). For quantitative analysis of Ca^2+^ activity recordings, an algorithm for automated identification and analysis of regions of interest (ROIs) in two-dimensional image sequences was used (Francis et al., 2012). Intracellular Ca^2+^ release was examined upon stimulation with the vasoconstrictor endothelin-I (ET-I) (0.1 μM and 1 μM) (Figures 2A, 2B, and S2C). Notably, NC-SMCs differentiated using protocols C and D showed lower responses to ET-I (0.1 μM, Figure 2A and 1 μM, Figure S2C) and had a considerably lower expression level of ET-I receptor (*EDNRA*), while expression of other receptors and ion channels was comparable (Figure S1E). NC-SMCs differentiated using protocols A, B, and E, as well as HBVSMCs, showed substantial variation in the number of Ca^2+^ events with a single or main peak (MP) and recurrent secondary peaks (SPs), while the vast majority of NC-SMCs differentiated using protocols C and D, as well as HBVPs, had only one MP (Figures 2B and 2C).

Video S1. Intracellular Ca^2+^ Release in NC-SMCs, Related to Figure 2

To describe and compare shapes of Ca^2+^ signals, we derived kinetic parameters of the average fluorescence intensity of ROIs normalized to baseline over time (F/F_0_). Time to peak, decay, and duration were measured at the half-maximum level (F/F_0_)_max_ (Figure 2D). Ca^2+^ kinetic parameters showed substantial heterogeneity in all vSMCs examined (Figure 2E). Distributions of time to peak and decay were positively skewed while the distribution of duration was close to Gaussian and the number of events within ROI was distributed exponentially. Bimodal histograms of time to peak in NC-SMCs differentiated using protocols C, D and HBVPs revealed subpopulations with slower and faster Ca^2+^ release and reuptake (Figure 2E).

**Figure 3:**
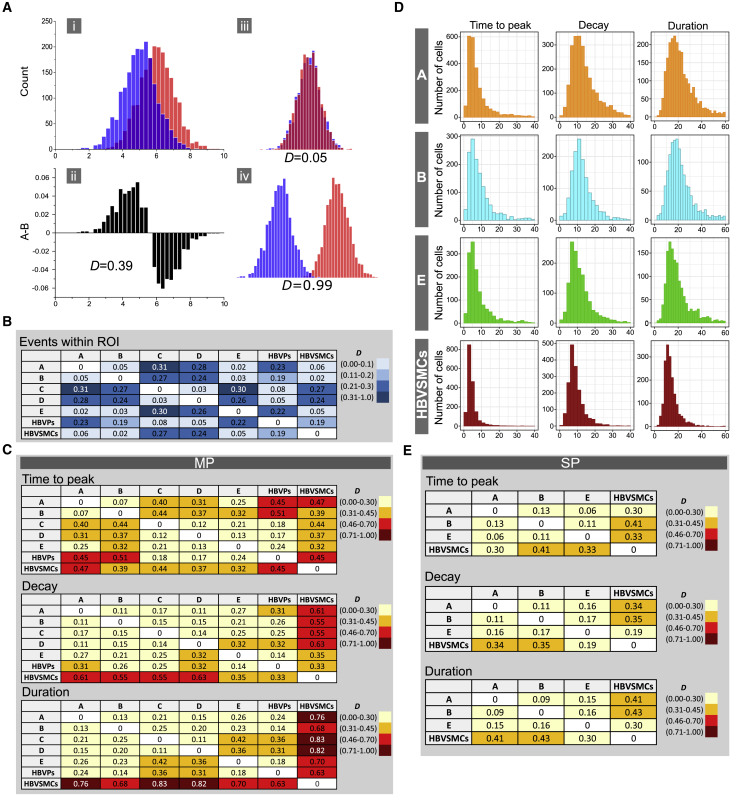
Comparison of Intracellular Ca^2+^ Release in NC-SMCs and Primary vSMCs (A) Schematic illustration of the method describing analysis of the divergence of two histograms: (i) two histograms shifted by 1 SD; (ii) subtraction of normalized histograms shifted by 1 SD and corresponding *D* value; (iii) example of largely overlapping histograms and corresponding *D* value; (iv) example of nearly non-overlapping histograms and corresponding *D* value. (B) Differences (*D* values) in a number of Ca^2+^ release events within each individual ROI evoked by stimulation of NC-SMCs with ET-I (0.1 μM) (differentiated using protocols A to E), HBVPs, and HBVSMCs. Legend indicates color coding depicting *D*-value ranges. (C) Differences (*D* values) in MP kinetic parameters (time to peak, decay, and duration) of Ca^2+^ release evoked by stimulation of NC-SMCs with ET-I (0.1 μM) (differentiated using protocols A to E), HBVPs, and HBVSMCs. Legends indicates color coding depicting *D*-value ranges. (D) Population histograms of SP parameters of Ca^2+^ release (time to peak, decay, and duration) in NC-SMCs (differentiated with protocols A, B, and E) and HBVSMCs. Data from three independent experiments are shown. (E) Differences (*D* values) in SP kinetic parameters (time to peak, decay, and duration) of Ca^2+^ release evoked by stimulation of NC-SMCs with ET-I (0.1 μM) (differentiated using protocols A, B, and E) and HBVSMCs. Legends indicate color coding depicting *D*-value ranges.

To compare population histograms quantitatively, we used a method reported previously that allows measurement of the difference between two populations (James et al., 2011). A schematic illustration of the method is shown in Figure 3A. The method is based on a simple metric that estimates the extent of divergence of two histograms reflected by a value *D*, which is close to zero if histograms are largely overlapping and equals 1 when histograms do not overlap at all (Figure 3A, Supplemental Experimental Procedures, and Equation 3). We classified ranges of *D* values into three categories that reflect the extent of histogram divergence for each type of measurement: events within ROI (low: *D =* 0.0–0.1; moderate: *D =* 0.11–0.2; high: *D =* 0.21–1.0) and kinetic parameters of Ca^2+^ peaks (low: *D =* 0.0–0.3; moderate: *D =* 0.31–0.45; high: *D =* 0.46–1.0). The rationale for setting different ranges for these types of measurement was based on corresponding intrapopulation *D* values between either technical replicates or independent biological experiments (data not shown).

When comparing the number of Ca^2+^ events within ROI, we obtained low *D* values between HBVPs and NC-SMCs differentiated with protocols C and D (*D =* 0.08 and *D =* 0.05, respectively) but moderate to high *D* values between HBVPs and NC-SMCs differentiated with protocols A, B, and E and HBVSMCs (*D =* 0.19–0.23) (Figure 3B). HBVSMCs showed low *D* values compared with NC-SMCs differentiated with protocols A, B, and E (*D =* 0.02–0.06) and moderate to high *D* values compared with NC-SMCs differentiated with protocol C and D and HBVPs (*D =* 0.19–0.27) (Figure 3B). Comparison of HBVSMCs with all NC-SMCs showed moderate to high *D* values of kinetic parameters of MP (time to peak: *D =* 0.32–0.47; decay: *D =* 0.35–0.63; duration: *D =* 0.68–0.83) (Figure 3C) but low to moderate *D* values of SPs in NC-SMCs differentiated with protocols A, B, and E (time to peak: *D =* 0.30–0.41; decay: *D* = 0.19–0.35; duration: *D* = 0.30–0.43) (Figures 3D and 3E). Predominantly low to moderate *D* values of kinetic parameters of MP were obtained on comparing HBVPs and NC-SMCs (time to peak: *D* = 0.17–0.51; decay: *D* = 0.14–0.32; duration: *D* = 0.14–0.36) (Figure 3C).

### Quantitative Assessment of Contraction in NC-SMCs and Primary vSMCs

**Figure 4:**
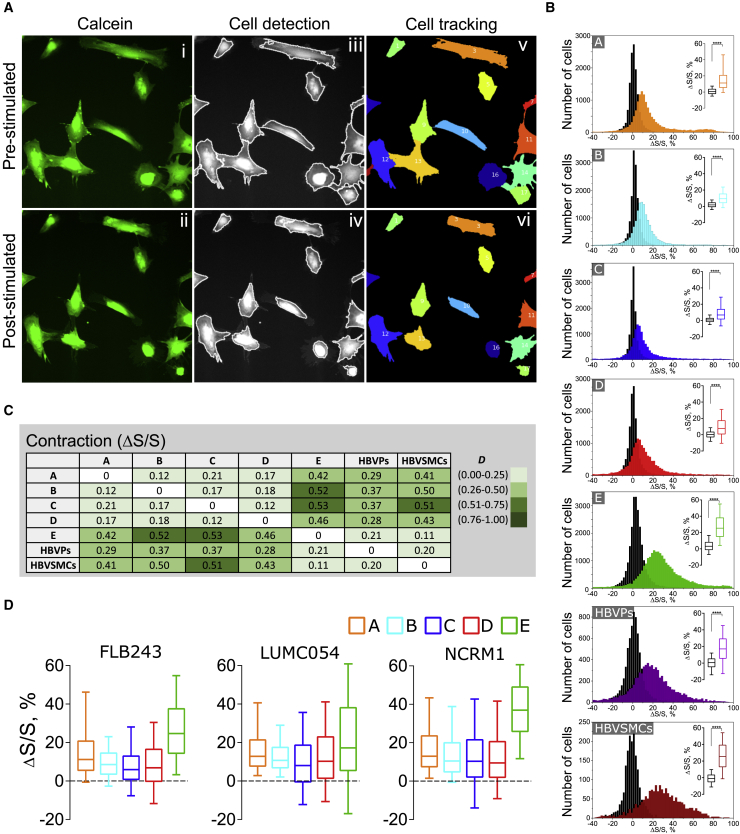
Quantitative Assessment of Contraction of NC-SMCs and Primary vSMCs (A) Representative images of NC-SMCs in a pre-stimulated (top row) and post-stimulated state after 30 min of ET-I addition (bottom row). (i, ii) fluorescent image of cells loaded with calcein; (iii, iv) overlapped image of cells and outlines after automated object identification; (v, vi) tracked objects depicting individual cells with a tracking number. (B) Distribution of relative cell-surface area change of NC-SMCs (differentiated with protocols A to E), HBVPs, and HBVSMCs upon control addition of B(P)EL medium (black histograms) and ET-I (0.1 μM) stimulation. Box plots indicate first, second (median), and third quartiles of relative surface area change upon control (in black) and after stimulation (in colors), and whiskers indicate 10^th^ and 90^th^ percentiles. Data from four (NC-SMCs) and two (HBVPs, HBVSMCs) independent experiments are shown. ^∗∗∗∗^p < 0.0001. (C) Differences (*D* values) in relative cell-surface area decrease of NC-SMCs (differentiated with protocols A to E), HBVPs, and HBVSMCs evoked by stimulation with ET-I (0.1 μM). Legend indicates color coding depicting *D*-value ranges. (D) Relative cell-surface area decrease of NC-SMCs (differentiated with protocols A to E) from FLB243, LUMC054, and NCRM1 hiPSC lines upon ET-I (0.1 μM) stimulation. Box plots indicate first, second (median), and third quartiles of relative surface area change, and whiskers indicate 10^th^ and 90^th^ percentiles. Data from four (FLB243) and one (LUMC054, NCRM1) independent experiments are shown.

To assess vSMC contraction, we developed an automated pipeline that included primary object identification and object tracking based on pixel intensity (Figure 4A and Video S2). This allowed image processing to be automated and tracking of changes in the surface area of individual cells, with unbiased processing of large numbers of cells captured in given fields of view. Individual cell contraction was calculated as relative change of cell-surface area (Δ*S*/*S*, in percent) (Supplemental Experimental Procedures; Equations 1 and 2) and plotted as population histograms. Contraction of NC-SMCs differentiated using protocols A to E, HBVPs, and HBVSMCs upon stimulation with vasoconstrictor ET-I or control stimulation (medium only, black histograms) was next evaluated (Figure 4B). Although we did observe fluctuations in cell-surface area upon control stimulation, the distributions had median values close to zero and a small dispersion, which was significantly different compared with stimulation with vasoconstrictor ET-I (0.1 μM) (p < 0.0001) (Figure 4B, box plots). Notably, NC-SMCs differentiated using protocols C and D showed the lowest relative cell-surface area decrease (Figure 4B). Stimulation with a higher concentration of ET-I (1 μM) did not increase contraction values (Figure S2D). We next compared population histograms of relative cell area decrease in all conditions, using analysis of difference described above (Figure 3A). We classified ranges of *D* values of contraction Δ*S*/*S* into three categories (low: *D* = 0.0–0.25; moderate: *D* = 0.26–0.50; high: *D* = 0.51–1.0) to reflect the extent of the differences. NC-SMCs differentiated using protocol E had the lowest difference compared with HBVSMCs (*D* = 0.11) and HBVPs (*D* = 0.21) among all NC-SMCs (Figure 4C) and showed high median relative cell-surface area decrease (Figure 4B, box plots). NC-SMCs differentiated using protocols A to D showed moderate differences compared with HBVPs (*D* = 0.28–0.37), moderate to high differences compared with HBVSMCs (*D* = 0.41–0.51), and low median relative cell-surface area decreases. Additionally, side-by-side assessment of contraction upon stimulation with ET-I (0.1 μM) in NC-SMCs differentiated using protocols A to E from three independent hiPSC lines showed reproducible decreases in relative cell-surface area, with NC-SMCs differentiated using protocol E being the most contractile (Figure 4D).

Video S2. Analysis of Contraction in NC-SMCs, Related to Figure 4

### Comparative Analysis of Intracellular Ca^2+^ Release and Contraction in NC-SMCs

To evaluate the robustness and functional reproducibility of the 12-day protocol A, we evaluated Ca^2+^ release and contraction of NC-SMCs differentiated from three independent hiPSC lines and compared this with cells derived using protocol B. Intracellular Ca^2+^ release in NC-SMCs differentiated using either protocol was examined upon stimulation with various vasoconstrictors, such as ET-I (0.1 μM), carbachol (Cch) (100 μM), and angiotensin II (Ang-II) (0.5 μM) (Figure S3A). All three agonists induced Ca^2+^ increases in a concentration-dependent manner (Figure S3B). Low *D* values of kinetic parameters (time to peak: *D* = 0.09–0.24; decay: *D* = 0.07–0.3; duration: *D* = 0.09–0.28) and low to moderate *D* values of number of events within ROI (*D* = 0.02–0.17) were obtained when measuring differences between NC-SMCs differentiated using protocols A and B (Figure S3C). Comparison of Ca^2+^ responses in NC-SMCs differentiated from independent hiPSC lines showed low to moderate *D* values of kinetic parameters of MP (time to peak: *D* = 0.11–0.27; decay: *D* = 0.16–0.43; duration: *D* = 0.15–0.44), kinetic parameters of SPs (time to peak: *D* = 0.07–0.26; decay: *D* = 0.11–0.33; duration: *D* = 0.14–0.34), and number of events within ROI (*D* = 0.02–0.2) (Figure S3D).

Differences in kinetic parameters of Ca^2+^ MP initiated by ET-I, Cch, and Ang-II were observed in NC-SMCs differentiated from the FLB243 hiPSC line (ET versus Cch: *D* = 0.13–0.41; ET versus Ang-II: *D* = 0.28–0.65; Cch versus Ang-II: *D* = 0.16–0.59), and to a lesser extent NC-SMCs differentiated from the NCRM1 hiPSC line (ET versus Cch: *D* = 0.18–0.37; ET versus Ang-II: *D* = 0.29–0.51; Cch versus Ang-II: *D* = 0.11–0.24) (Figure S3E). NC-SMCs differentiated from the LUMC054 hiPSC line showed comparable kinetic parameters of Ca^2+^ MP by various vasoconstrictors (ET versus Cch: *D* = 0.2–0.3; ET versus Ang-II: *D* = 0.14–0.24; Cch versus Ang-II: *D* = 0.16–0.24) (Figure S3E). These variations were not observed in SPs in all hiPSC lines (ET versus Cch: *D* = 0.06–0.3; ET versus Ang-II: *D* = 0.05–0.28; Cch versus Ang-II: *D* = 0.07–0.34) (Figure S3E). Importantly, NC-SMCs differentiated with protocol B showed higher variability in number of events within ROI in contrast to protocol A (protocol A: *D* = 0.02–0.14; protocol B: *D* = 0.05–0.25) (Figure S3E).

Relative cell-surface area decrease upon stimulation with ET-I (0.1 μM) in NC-SMCs differentiated from three independent hiPSC lines with protocols A or B and showed low *D* values (protocol A: *D* = 0.12–0.18; protocol B: *D* = 0.13–0.16) (Figures S4A–S4C). NC-SMCs differentiated using protocol A showed slightly greater relative cell-surface area decreases (Figure S4A).

## Discussion

Since vSMCs are highly heterogeneous and may differ in developmental origin, we focused on the neural crest population, which has been shown previously to be a major source of vSMCs in the cerebral vasculature. Accordingly, we chose HBVPs and HBVSMCs as a primary human perivascular cell type of neural crest origin as a comparator. We successfully obtained NCCs and derivative NC-SMCs from three independent hiPSC lines using methods based on previously published protocols. We analyzed large ensembles of these NC-SMCs in Ca^2+^ and contraction assays and compared their responses with HBVP and HBVSMC outputs.

We observed that NC-SMCs differentiated with protocols A, B, or E were most like HBVSMCs in the Ca^2+^ assay in exhibiting SPs, albeit with slower kinetics of Ca^2+^ release and reuptake. NC-SMCs differentiated with protocols A and E showed the highest relative cell-surface area decrease upon contraction among all hiPSC-derived NC-SMCs. Although NC-SMCs differentiated with protocol E appeared more similar to HBVSMCs based on Ca^2+^ release and contractile behavior, their differentiation took much longer and included high concentrations of fetal bovine serum while protocol A was shorter and serum free. Thus, when contractile phenotypes are required, we concluded that protocol A is fit for purpose. On the other hand, NC-SMC differentiation with protocols C and D results in cells functionally closer to HBVPs, although expression of contractile SMC markers was higher compared with HBVPs. Importantly, these assays produced a wide range of detailed information on the vSMC response to stimuli, providing useful information to characterize the hiPSC-derived cells, select the most appropriate protocol, and thus ensure that the vSMCs are fit for purpose in downstream assays.

In summary, we have provided an unambiguous method for functional analysis of hiPSC-vSMCs that provides multiple parameters to accurately access phenotype in the overall cell population, including heterogeneity in Ca^2+^ and contractile responses across NC-SMCs differentiated from different hiPSC lines. This overcomes one major limitation of the efficient use of vSMCs in current studies, namely the lack of specific protein or genetic markers that distinguish perivascular cell types. As many disease states involve switches in contractile phenotype and differential responses to vasoconstrictor stimuli, we expect the methodology to provide a robust approach for quantification and specification of vSMC functionality.

## Experimental Procedures

Full details are provided in Supplemental Experimental Procedures.

### hiPSC Maintenance

hiPSCs were cultured on Matrigel-coated plates in mTeSR-1 or recombinant vitronectin-coated plates in TeSR-E8 all from STEMCELL Technologies, according to the manufacturer's instructions.

### Data Analysis

#### Intracellular Ca^2+^ Release

Images were processed using a freely available plugin “LC Pro” for ImageJ (Francis et al., 2012). Free open-source CellProfiler software (Carpenter et al., 2006) was used to determine the total number of cells in a field of view. Output data were analyzed using a customized R-based script. MP was defined as the first Ca^2+^ release that took place within ∼100 s after administration of the vasoconstrictor. SPs were defined as Ca^2+^ events after MP if two or more peaks were detected within a given ROI.

#### Contraction

Images were processed using a customized pipeline that included automated cell identification and tracking using CellProfiler. Output data were analyzed using a customized R-based script. Contraction was defined as a percentage of cell-surface area decrease (Equations 1 and 2):

(Equation 1) $${\left(\frac{\Delta S}{S}\right)}_{\text{control}}^{i}=\frac{S_{1}^{i}-S_{2}^{i}}{S_{1}^{i}}\text{,}$$

(Equation 2) $${\left(\frac{\Delta S}{S}\right)}_{\text{drug}}^{i}=\frac{S_{2}^{i}-S_{3}^{i}}{S_{2}^{i}}\text{,}$$

where *i* is the tracking index number of a single cell; $S_{1}^{i}$, $S_{2}^{i}$, and $S_{3}^{i}$are cell-surface area measured from pre-stimulated, negative control, and post-stimulated states, respectively.

### Statistical Analysis

All statistical analyses were performed using GraphPad Prism 7 software. The data are reported as mean ± SD. D-metrics was used to measure the difference between two population histograms as a discrete estimator of Kullback-Leibler divergence and calculated using Equation 3:

(Equation 3) $$D=\frac{1}{2}\sum_{i=1}^{n}\left|A_{i}-B_{i}\right|\text{,}$$

where *n* is total number of bins.

## Author Contributions

O.V.H. designed and performed research, analyzed and interpreted results, and wrote the manuscript; A.C. analyzed and interpreted results and edited the manuscript; F.E.v.d.H. performed real-time PCRs; C.L.M. designed the research, interpreted results, and edited the manuscript; V.V.O. designed the research, interpreted results, and wrote the manuscript.

## Supplemental Information

Document S1. Supplemental Experimental Procedures and Figures S1–S4

Document S2. Article plus Supplemental Information
