# Supplementary material for: Quantitative Analysis of Intracellular Ca2+ Release and Contraction in hiPSC-Derived Vascular Smooth Muscle Cells
Source: Stem Cell Reports. 2019 Mar 7;12(4):647–56. doi: 10.1016/j.stemcr.2019.02.003 (PMC6449838; doi:10.1016/j.stemcr.2019.02.003)
Supplement: Document S1. Supplemental Experimental Procedures and Figures S1–S4 [file mmc1.pdf]

Stem Cell Reports, Volume *12*

Supplemental Information

Quantitative Analysis of Intracellular Ca^{2+} Release and Contraction in hiPSC-Derived Vascular Smooth Muscle Cells

Oleh V. Halaidych, Amy Cochrane, Francijna E. van den Hil, Christine L. Mummery, and Valeria V. Orlova

Supplemental Information

Quantitative analysis of intracellular Ca^{2+} release and contraction in hiPSC-derived vascular smooth muscle cells

Oleh V. Halaidych, Amy Cochrane, Francijna E. van den Hil, Christine L. Mummery and Valeria V. Orlova

Supplemental Figures and Legends

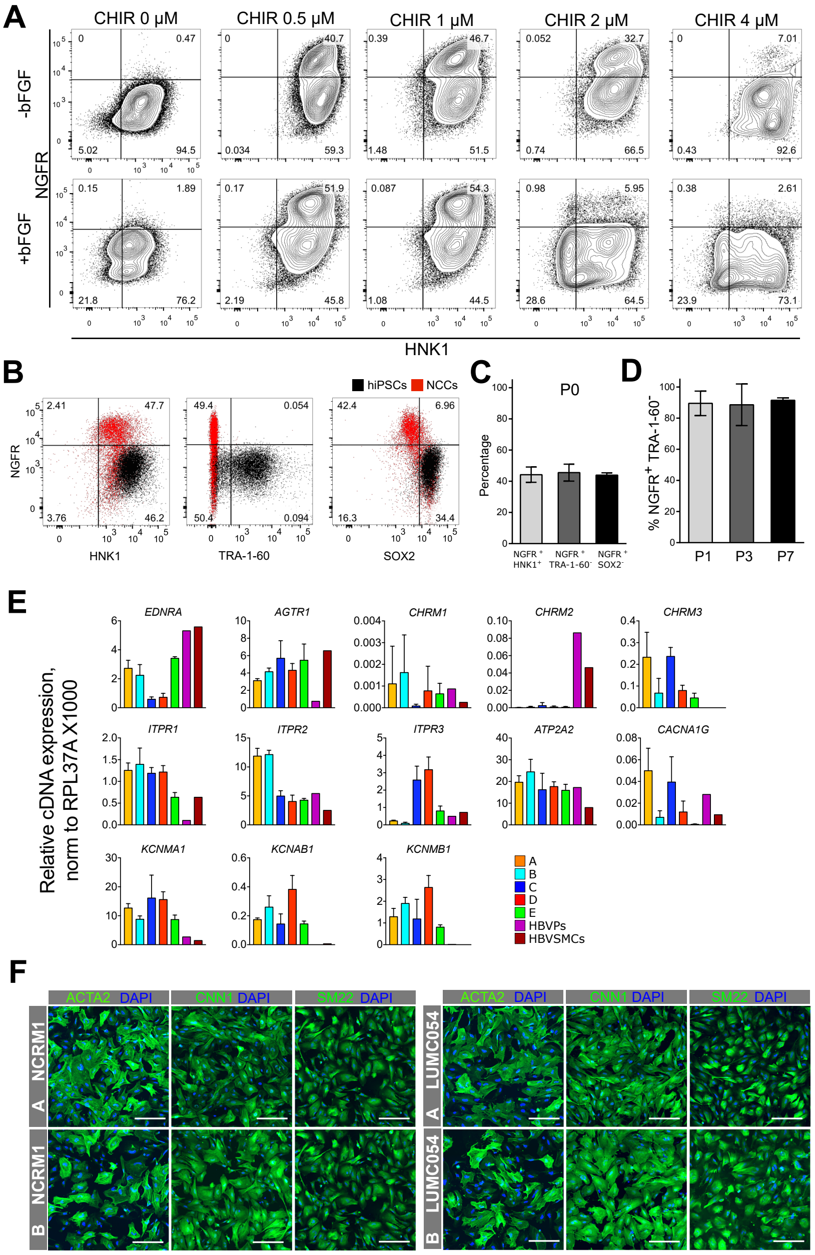

Figure S1. Related to Figure 1. Differentiation of NCCs and NC-SMCs from hiPSCs. (A) Representative FACS plots showing NGFR, HNK1 expression at day 12 of neural crest differentiation using indicated concentrations of CHIR without (top row) and with (bottom row) bFGF supplementation. **(B)** Representative FACS plots showing NGFR, HNK1, TRA-1-60, SOX2 expression at day 12 of neural crest differentiation (in red) and non-differentiated hiPSCs (in black). **(C)** Percentage of NGFR$^+$/HNK1$^+$, NGFR$^+$/TRA-1-60$^-$, NGFR$^+$/SOX2$^-$ cells at day 12 of neural crest differentiation. Bars are shown as mean ± SD from two independent differentiation experiments. **(D)** Percentages of NGFR$^+$/TRA-1-60$^-$ NCCs at passage 1, passage 3 and passage 7 (P1, P3 and P7). Bars are shown as mean ± SD of three independent differentiation experiments. **(E)** RT-PCR analysis of relative gene expression of *EDNRA, AGTR1, CHRM1, CHRM2, CHRM3, ITPR1, ITPR2, ITPR3, ATP2A2, CACNA1G, KCNMA1, KCNAB1, KCNMB1* in NCCs, NC-SMCs differentiated using protocols A, B, C, D, E, HBVPs and HBVSMCs. Bars represented as mean ± SD from three independent experiments and normalized to housekeeping gene *RPL37A* (X1000). **(F)** Representative immunofluorescent images showing expression of ACTA2, CNN1, SM22 (in green) and DAPI (in blue) in NC-SMCs differentiated using protocols A and B from NCRM1 and LUMC054 hiPSCs lines. Scale bar, 100 µm.

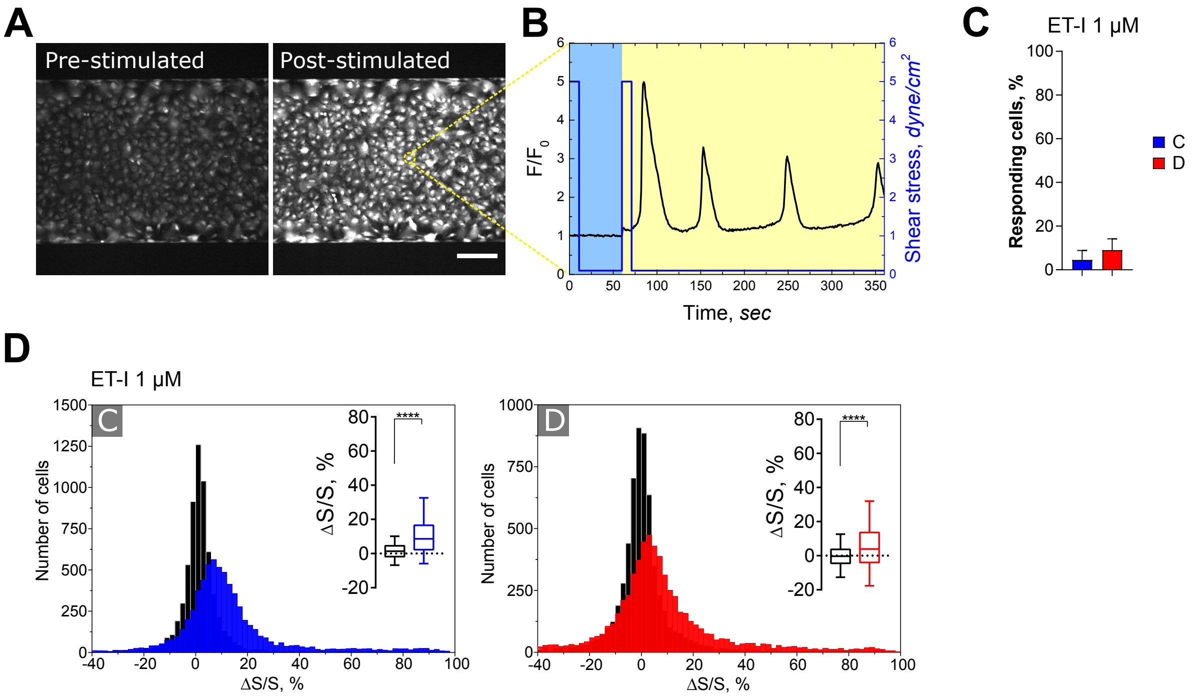

Figure S2. Related to Figure 2 and Figure 4. Quantitative Assessment of Intracellular Ca^{2+} release and Contraction in NC-SMCs and Primary vSMCs. (A) Cumulative time-lapse image of intracellular Ca^{2+} fluorescence in NC-SMCs in pre- and post- stimulated states. Scale bar, 200 µm **(B)** Overlay graph depicting normalized fluorescence intensity F/F_0 of intracellular Ca^{2+} in a representative cell (black trace) and fluid flow shear stress (blue trace) over time. Blue and yellow background depict time of pre- and post-stimulation with vasoconstrictor. **(C)** Fraction of responding cells stimulated with ET-I (1 µM) detected with automated image processing above statistical noise ($p<0.01$). NC-SMCs differentiated with protocol C (in dark blue) and D (in red) are depicted. Data from two independent experiments are shown. **(D)** Distribution of relative cell surface area change upon control addition of B(P)EL medium (black histograms) and ET-I (1 µM) stimulation of NC-SMCs differentiated with protocol C (dark blue histograms) and D (red histograms). Boxplots indicate first, second (median), third quartiles of relative surface area change upon control (in black) and after stimulation (in colors) and whiskers indicate 10th and 90th percentiles. Data from two independent experiments are shown. **** $p<0.0001$.

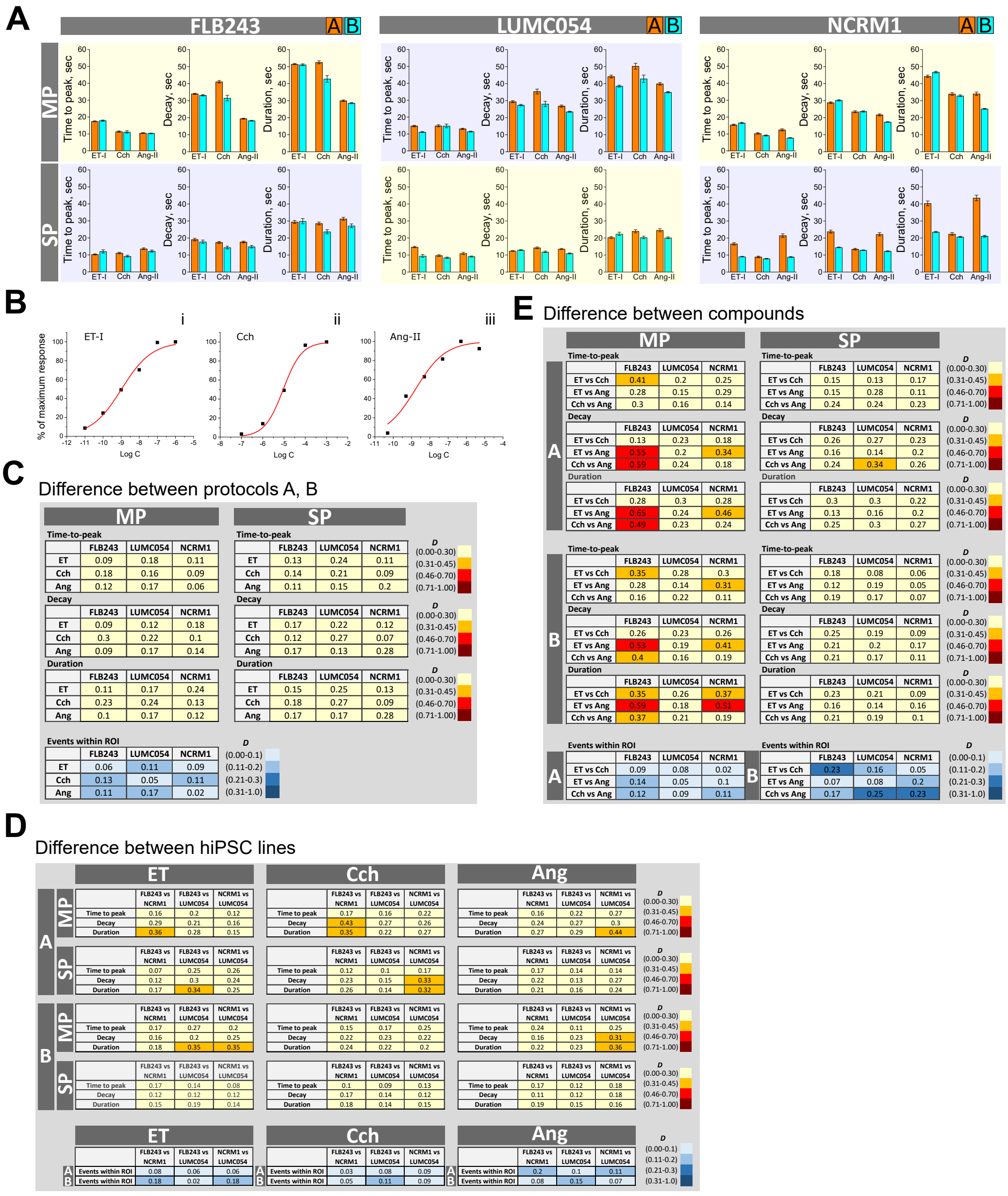

Figure S3. Related to Figure 2 and Figure 3. Comparative Analysis of Intracellular Ca^{2+} release in NC-SMCs. (A) Mean parameters of MP and SP Ca^{2+} release evoked by different agonists (ET-I (0.1 µM), Cch (100 µM), Ang-II (0.5 µM)) in NC-SMCs differentiated with protocol A and B from FLB243, LUMC054 and NCRM1 hiPSCs lines. Bars are shown as mean ± SE from three independent experiments. **(B)** Dose-response curves of Ca^{2+} release in NC-SMCs (Protocol A, FLB243) after stimulation with (i) ET-I (0.01 nM; 0.1 nM; 1 nM; 10 nM; 0.1 µM; 1 µM), (ii) Cch (0.1 µM; 1 µM; 10 µM; 100 µM, 1 mM), (iii) Ang-II (0.05 nM; 0.5 nM; 5 nM; 50 nM; 0.5 µM; 5 µM). Percentage of responding cells (black dots) out of maximal response for each drug is shown. Data fitting was performed using "sigmoidal" function (red curves). **(C)** Differences (*D*-values) in parameters of Ca^{2+} release (time to peak, decay, duration, number of events within ROI) between responses triggered in NC-SMCs differentiated with protocols A and B. Legends indicate the color-coding depicting *D*-value ranges. **(D)** Differences (*D*-values) in parameters of Ca^{2+} release (time to peak, decay, duration, number of events within ROI) between responses in NC-SMCs differentiated from three independent hiPSCs lines. Legend indicates the color-coding depicting *D*-value ranges. **(E)** Differences (*D*-values) in parameters of Ca^{2+} release (time to peak, decay, duration, number of events within ROI) between responses triggered by different agonists (ET-I (0.1 µM), Cch (100 µM), Ang-II (0.5 µM)) in NC-SMCs differentiated with protocols A and B. Legends indicate the color-coding depicting *D*-value ranges.

A

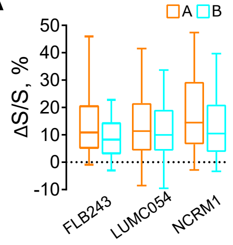

B

Contraction (ΔS/S)

	A	B
FLB243 vs NCRM1	0.18	0.16
FLB vs LUMC054	0.12	0.16
NCRM1 vs LUMC054	0.16	0.13

D
(0.00-0.25)
(0.26-0.50)
(0.51-0.75)
(0.76-1.00)

C

Contraction (ΔS/S)

	A vs B
FLB243	0.12
NCRM1	0.13
LUMC054	0.16

D
(0.00-0.25)
(0.26-0.50)
(0.51-0.75)
(0.76-1.00)

Figure S4. Related to Figure 4. Comparative Analysis of Contraction in NC-SMCs. (A) Relative cell surface area decrease upon ET-I stimulation of NC-SMCs differentiated with protocol A (orange bar), B (light blue bar) from FLB243, LUMC054 and NCRM1 hiPSCs lines. Boxplots indicate first, second (median), third quartiles of relative surface area change upon stimulation with ET-I 0.1 μM and whiskers indicate 10th and 90th percentiles. Data from four (FLB243) and two (LUMC054, NCRM1) independent experiments are shown. **(B)** Differences (*D*-values) in relative cell surface area decrease evoked by stimulation with ET-I 0.1 μM between NC-SMCs differentiated from FLB243, NCRM1 and LUMC054 hiPSCs lines. Legend indicates the color-coding depicting *D*-value ranges. **(C)** Differences (*D*-values) in relative cell surface area decrease evoked by stimulation with ET-I 0.1 μM between NC-SMCs differentiated with protocol A and B. Legend indicates the color-coding depicting *D*-value ranges.

Supplemental Experimental Procedures

hiPSC lines and maintenance

The following hiPSC lines were used: NCRM1 (NIH Center for Regenerative Medicine (NIH CRM), obtained from RUDCR Infinite Biologics at Rutgers University), LUMC054 (LUMC0054iCTRL, additional information available in public databases: http://hpscreg.eu/cell-line/LUMCi001-A)) (Halaidych et al., 2018) and FLB243 (SFLB6) (Zhang et al., 2014). The NCRM1 hiPSC line was maintained on Vitronectin-coated plates in TeSR-E8 medium (StemCell Technologies, 05940) and passaged once a week using Gentle Cell Dissociation Reagent (StemCell Technologies, 07174). LU054 and FLB243 lines were cultured in growth factor reduced Matrigel-coated plates (BD Biosciences, 354230) in mTeSR-1 medium (StemCell Technologies, 05850). hiPSC lines maintained in mTeSR-1 medium were routinely passaged mechanically once a week using Dispase solution 1 mg/mL (Gibco, 17105-041).

NC differentiation

hiPSC colonies were passaged and kept in hiPSC maintenance medium. After 2 days, the medium was changed to NC differentiation medium consisting of BPEL (without PVA) (Ng et al., 2008) supplemented with 10 µM SB431542 (Tocris Bioscience, 1614), 1 µM CHIR99021 (Tocris Bioscience, 4423), 10 ng/mL bFGF (Miltenyi Biotec, 130-093-842). Cells were refreshed every 2 days and kept in NC differentiation medium for 10-12 days. After 10-12 days NC cells (NCCs) were passaged with 1xTrypLE Select (Gibco, 12563029) and plated in 1:4 ratio on Matrigel-coated plates. NCCs were cryopreserved at passage number 3 (P3) in CryoStor CS10 medium (StemCell Technologies, 07930).

SMC differentiation

NCCs were plated at $3x10^4$ cells/cm^2 seeding density on 0.1% Gelatin (Sigma-Aldrich, G1890) coated plates in SMC differentiation media consisting of BPEL (without PVA) (Ng et al., 2008) supplemented with 2 ng/mL TGF-β3 (a generous gift of Kenneth K. Iwata, OSI Pharmaceuticals) and 10 ng/mL PDGF-bb (PeproTech, 100-14B) for 12 days (Protocol A) or 6 days (Protocol B). Protocol B was initiated at day 6 when the medium was changed to B(P)EL medium supplemented with 1 ng/mL TGF-β3 and 0.5% FBS (Gibco, 10270-106) for additional 6 days. Protocol C was a continuation of Protocol A for another 18 days (30 days in total). Protocols D and E were initiated after day 12 of differentiation with Protocol A by following differentiation for 18 days in B(P)EL supplemented with 1) 1 ng/mL TGF-β3 and 0.5% FBS or 2) 10% FBS respectively. When reaching 100% confluency cells were passaged in a 1:4 splitting ratio. At day 12 (Protocols A, B) or day 30 (Protocols C, D, E) cells were refreshed with supplement-free B(P)EL for at least 24 h before use in functional assays.

HBVPs and HBVSMCs were purchased from ScienceCell. HBVPs were cultured in Pericyte Medium (ScienceCell, 1201) supplemented with Pericyte Growth Supplement (ScienceCell, 1252) and 2% FBS. HBVSMCs were cultured in Smooth Muscle Cell Medium (ScienceCell, 1101) supplemented with Smooth Muscle Growth Supplement (ScienceCell, 1152) and 2% FBS.

Flow cytometry analysis

Cells were dissociated with 1xTrypLE Select and washed once with FACs buffer containing 10% FBS, and once with FACs buffer. The following surface antibodies were used for the FACS staining: NGFR-BV421 (BD Biosciences, 562562, 1:100), HNK1-FITC (BD Biosciences, 555619, 1:20), TRA-1-60-PE (Miltenyi, 130-100-347, 1:20), PDGFRα-BV421 (BD Biosciences, 562799, 1:200), PDGFRβ-PE (BD Pharmingen, 558821, 1:20), CD146-FITC (DB Pharmingen, 560846, 1:20), CD73-PE (BD Pharmingen, 550257, 1:20), CD105-PE-Vio-770 (Miltenyi, 130-099-889, 1:10), CD44-FITC (Biolegend, 338804, 1:200), CD90-PE-Vio-770 (Miltenyi, 130-099-296, 1:10), NG2-PE (R&D Systems, FAB2585P, 1:20). For intracellular labelling with SOX2-A488 (eBiosciences, 53-9811-80, 1:50) and MYH11 (Sigma-Aldrich, M7786, 1:200) the cells were fixed and permeabilized using BD Cytofix/Cytoperm kit (BD Biosciences, 554714). Analysis of samples was performed on the MACSQuant VYB (Miltenyi Biotec, 130-096-116) equipped with the following lasers/filters: Violet/405 nm BV421: 450/50, Blue/488 nm FITC, A488: 525/50, Yellow/561 nm PE: 586/15, Yellow/561 nm PE-Vio-770: 750 nm LP.

Immunofluorescence staining

NCCs and SMCs were grown in 96 well plates (Corning, 353219) to a confluent monolayer. Cells were fixed with 4% PFA, permeabilized with 0.05% TX-100 (Sigma). the following antibodies were used:

Primary antibodies					
Protein target	Company	Cat. number	Host species	Clonality	Dilution
SOX10	Abcam	ab108408	Rabbit	polyclonal	1:200
SOX9	EMD Milipore	AB5535	Rabbit	polyclonal	1:200
TFAP2A	Genetex	GTX62588	Rabbit	monoclonal	1:200
SOX2	eBiosciences	53-9811-80	Rat	monoclonal	1:200
ACTA2	Sigma	1A4	Mouse	monoclonal	1:200
MYH11	Sigma	M7786	Mouse	monoclonal	1:200
SM22	Abcam	ab14106	Rabbit	polyclonal	1:400
CNN1	Sigma	C2687	Mouse	monoclonal	1:200

Immunofluorescence images were acquired using BD Pathway 855 (BD Biosciences, 641760) or EVOS FL AUTO2.

Quantitative real-time polymerase chain reaction (RT-PCR)

Total RNA was extracted from NCCs or NC-SMCs using the NucleoSpin® RNA II Kit (Macherey-Nagel) combined with Ambion® TURBO™ DNase treatment (Life Technologies, AM1907) and 500 ng RNA was used to generate cDNA using the iScript cDNA Synthesis Kit (Bio-Rad). qPCR was performed using the CFX384 Touch™ Real-Time PCR Detection System (Bio-Rad) and data was analysed with Bio-Rad CFX Manager 3.0 software. For each 10 µL qPCR reaction we used 2.5 ng cDNA with 5 µM FW primer, 5 µM RV primer; 5 µL iQ™ SYBR Green Supermix (Bio-Rad). Samples were denatured for 3 min at 95 °C followed by 45 cycles of 15 s at 95 °C, 30 s at 60 °C and 45 s at 72 °C. Melt-curve analysis was performed directly after the amplification protocol under the following conditions: 10 s denaturation at 95 °C and 0.5 °C increments of 5 s from 65 °C to 95 °C. The following primers were used for qPCR:

**Primer name**	**Sequence (FW primer)**	**Sequence (RV primer)**	**Size (bp)**
RPL37A	5’-GTGGTTCCTGCATGAAGACAGTG-3’	5’-TTCTGATGGCGGACTTTACCG-3’	84
CNN1	5’-TCATCAAGGCCATCACCAAGT-3’	5’-AGGGTGGACTGCACCTGTGTA-3’	100
SM22	5’-CGAAGTGCAGTCCAAAATCGAGAA-3’	5’-AATCACGCCATTCTTCAGCCAGAC-3’	148
MYH11	5’-GGCAACGCCAAAACAGTGA-3’	5’-TCAATGTTGGCTCCCACGAT-3’	101
SMA	5’-GTGATCACCATCGGAAATGAA-3’	5’-TCATGATGCTGTTGTAGGTGGT-3’	112
TFAP2A	5’-TCAAGTACGAGGACTGCGAG-3’	5’-AGGGAGATTGACCTACAGTGC-3’	90
PAX3	5’-GACTTGGAGAGGAAGGAGGC-3’	5’-CTTCATCTGATTGGGGTGCT-3’	100
SOX9	5’-GCTCTGGAGACTTCTGAACGA-3’	5’-CCGTTCTTCACCGACTTCCT-3’	132
SOX10	5’-CCTTCATGGTGTGGGCTC-3’	5’-CGCTTGTCACTTTCGTTCAG-3’	124
EDNRA	5’-TCGGGTTCTATTTCTGTATGCCC-3’	5’-TGTTTTTGCCACTTCTCGACG-3’	143
AGTR1	5’-ATTTAGCACTGGCTGACTTATGC-3’	5’-CAGCGGTATTCCATAGCTGTG-3’	76

CHRM1	5'-GGTGGGACATGCCAATCACTG-3'	5'-AGTGTTCATGGTGGCTAGGTG-3'	241
CHRM2	5'-GACTCCTCGCTCCTTCAAGC-3'	5'-AACCTGTCGCTGGTTTCGC-3'	72
CHRM3	5'-GCCTGACATCTGGTCCACTC-3'	5'-CATCGGAGGGGCTGTGTATC-3'	204
ITPR1	5'-GCGGAGGGATCGACAAATGG-3'	5'-TGGGACATAGCTTAAAGAGGCA-3'	133
ITPR2	5'-GCGTGTGTCCTTGGATGCT-3'	5'-CACCCTGGGTTATCAAGAAGC-3'	177
ITPR3	5'-CCAAGCAGACTAAGCAGGACA-3'	5'-ACACTGCCATACTTCACGACA-3'	142
ATP2A2	5'-ACAATGGCGCTCTCTGTTCT-3'	5'-ATCCTCAGCAAGGACTGGTTT-3'	83
CACNA1G	5'-ACACTTGGAACCGGCTTGAC-3'	5'-AGCACACGGACTGTCCTGA-3'	106
KCNMA1	5'-CGGACGCTCAAGTACCTGTG-3'	5'-AGCCATTGTTAATCTTCTGGGC-3'	79
KCNAB1	5'-GCAAATCGACCGGACAGTAAC-3'	5'-GCCATGCCTTGGTTTATCACAT-3'	77
KCNMB1	5'-CAAGAAGGTGCCCCAGTACC-3'	5'-GGGCCGTCTGGTAATTGTCC-3'	146

Assessment of intracellular Ca^{2+}

Differentiated NC-SMCs were passaged to make a single cell suspension and seeded into a microfluidic biochip (Vena8 Endothelial+, Cellix Ltd). Biochip channels were coated with 50 µg/mL bovine fibronectin (Sigma) at +37 °C for at least 1 h. Cells were seeded into 8 individual channels at density 10^4 cells/µL by injecting 6 µL of the suspension per each channel. Biochips were kept in a CO_2 incubator for 15-20 min. When cells started to attach and spread medium was added in all reservoirs of the biochips (100 µL per reservoir). Cells were kept in a CO_2 incubator for 3-4 h before functional analysis. Single channel perfusion was enabled by a PC-controlled syringe pump (Mirus Evo Nanopump, Cellix). Negative pressure was applied to the outlet end of the channel to introduce flow and pull in medium present in the inlet reservoir. The flow profile consisted of two sequential parts: 1) fast 5 dyne/cm^2 for 20 sec; 2) slow 0.1 dyne/cm^2 for the rest of the imaging experiment. Channels were loaded with a solution of calcium sensitive dye Fluo-4-AM (Ex/Em=494/506 nm, Invitrogen F14201) for 30 min at 37°C. After staining cells were perfused with dye-free BPEL medium for 5-10 min to wash the channel. The biochip was placed into a live imaging chamber (+37 °C, 5% CO_2, humidified) mounted on a Leica AF6000 microscope. We used an electron-multiplying charge-coupled device camera (Hamamatsu C9100) for time-lapse image acquisition. Image sequences of fluorescence were captured at 1 or 2 frames per second using a 10x objective with 2x2 binning (spatial resolution: 2.28 µm/pix). First, basal fluorescence activity upon B(P)EL medium flow was captured. Then image capturing was paused and the inlet reservoir was filled with vasoactive compound diluted in B(P)EL medium. Flow was applied again and simultaneous image capturing was continued.

Assessment of contraction of NC-SMCs and primary vSMCs

Differentiated NC-SMCs or primary HBVPs and HBVSMCs were passaged as single cells and plated in a 96 well plate at density ~10^4 cells/cm^2 in B(P)EL medium and kept in a CO_2 incubator overnight before functional analysis. Cells were loaded with 2 µM Calcein AM (Ex/Em=494/517 nm, Invitrogen L3224) for 30 min in a live imaging chamber (37°C, 5% CO_2, humidified). After the staining cells were gently washed with B(P)EL medium before assessment of the contraction. Series of images of Calcein fluorescence were captured using a Leica AF6000 microscope with a 10x objective and 4x4 automated stitching. First, the basal state of cells was acquired. Then a negative control was obtained by adding B(P)EL medium and fluorescence was acquired after 30 min. Finally, cells were stimulated with ET-I at a final concentration of 0.1 µM and fluorescence was acquired after 30 minutes.

Supplemental References

Halaidych, O.V., Freund, C., van den Hil, F., Salvatori, D.C.F., Riminucci, M., Mummery, C.L., Orlova, V.V., 2018. Inflammatory Responses and Barrier Function of Endothelial Cells Derived from Human Induced Pluripotent Stem Cells. STEMCR 10, 1642–1656. doi:10.1016/j.stemcr.2018.03.012

Ng, E.S., Davis, R., Stanley, E.G., Elefanty, A.G., 2008. A protocol describing the use of a recombinant protein-based, animal product-free medium (APEL) for human embryonic stem cell differentiation as spin embryoid bodies. Nature Protocols 3, 768–776. doi:10.1038/nprot.2008.42

Zhang, M., D'Aniello, C., Verkerk, A.O., Wrobel, E., Frank, S., Ward-van Oostwaard, D., Piccini, I., Freund, C., Rao, J., Seebohm, G., Atsma, D.E., Schulze-Bahr, E., Mummery, C.L., Greber, B., Bellin, M., 2014. Recessive cardiac phenotypes in induced pluripotent stem cell models of Jervell and Lange-Nielsen syndrome: disease mechanisms and pharmacological rescue. Proc. Natl. Acad. Sci. U.S.A. 111, E5383–92. doi:10.1073/pnas.1419553111
